# Evaluating Thera-101 as a Low-Volume Resuscitation Fluid in a Model of Polytrauma

**DOI:** 10.3390/ijms232012664

**Published:** 2022-10-21

**Authors:** Jessica Stukel Shah, Joseph Macaitis, Bridney Lundquist, Brian Johnstone, Michael Coleman, Michelle A. Jefferson, Jacob Glaser, Annette R. Rodriguez, Sylvain Cardin, Heuy-Ching Wang, Alexander Burdette

**Affiliations:** 1 Naval Medical Research Unit San Antonio, Fort Sam Houston, San Antonio, TX 78234, USA; 2 Theratome Bio, Inc., Indianapolis, IN 46202, USA; 3 Air Force Research Laboratory, 711th Human Performance Wing, Airman Systems Directorate, Bioeffects Division, Veterinary Science Branch, San Antonio, TX 78234, USA

**Keywords:** secretome, cell-based therapy, trauma, neuroprotection, organ damage

## Abstract

Traumatic brain injury (TBI) and hemorrhage remain challenging to treat in austere conditions. Developing a therapeutic to mitigate the associated pathophysiology is critical to meet this treatment gap, especially as these injuries and associated high mortality are possibly preventable. Here, Thera-101 (T-101) was evaluated as low-volume resuscitative fluid in a rat model of TBI and hemorrhage. The therapeutic, T-101, is uniquely situated as a TBI and hemorrhage intervention. It contains a cocktail of proteins and microvesicles from the secretome of adipose-derived mesenchymal stromal cells that can act on repair and regenerative mechanisms associated with poly-trauma. T-101 efficacy was determined at 4, 24, 48, and 72 h post-injury by evaluating blood chemistry, inflammatory chemo/cytokines, histology, and diffusion tensor imaging. Blood chemistry indicated that T-101 reduced the markers of liver damage to Sham levels while the levels remained elevated with the control (saline) resuscitative fluid. Histology supports the potential protective effects of T-101 on the kidneys. Diffusion tensor imaging showed that the injury caused the most damage to the corpus callosum and the fimbria. Immunohistochemistry suggests that T-101 may mitigate astrocyte activation at 72 h. Together, these data suggest that T-101 may serve as a potential field deployable low-volume resuscitation therapeutic.

## 1. Introduction

Poly-trauma, including hemorrhage and traumatic brain injury (TBI), is a leading cause of morbidity and mortality in both the civilian and military settings. In the civilian setting, over 26% of trauma patients with a severe brain injury also experience hemorrhagic shock [1]. In the military setting, the incidence rate of warfighters that sustained brain injuries plus concurrent hemorrhagic shock is closer to 80% [1,2,3]. Together, these injuries comprise a major focus that necessitates improved therapeutics from the point of injury through prolonged field care to enhance the repair and recovery of damaged organs and tissues.

It is known that TBI deteriorates outcomes and increases mortality in patients also experiencing hypotension and shock [4,5]. A potential reason for this increase may be that poly-trauma with TBI and hemorrhage results in metabolic destabilization, acidosis, inflammation, organ injury and failure, neurological dysfunction, and neuronal death [6,7]. Thus, approaches for metabolic and tissue stabilization to enable prolonged pre-hospital survivability are necessary.

The recommended resuscitation guidelines from the Brain Trauma Foundation for patients with TBI calls for a systolic blood pressure (SBP) > 100 mm Hg for patients aged 50–69, and >110 mm Hg for patients 15–49 years old and >70 years old [8]. The 2020 Tactical Combat Casualty Care Guidelines for Medical Personnel calls for maintaining SBP of 100–110 mm Hg in a casualty with an altered mental status due to suspected TBI. The required focus on brain protection using aggressive fluid resuscitation can produce deleterious effects, including end organ damage and induction of systemic inflammatory response syndrome, which lessens the overall recovery from poly-trauma [9,10]. Unfortunately, no therapeutic strategy exists to target the numerous repair and regeneration cellular pathways required following poly-trauma.

Mesenchymal stromal cell (MSC)-based therapeutics represent a promising strategy for addressing combat-related poly-traumatic injuries [11,12]. It is increasingly recognized that the MSC secretome, also termed paracrine factors or conditioned medium, is responsible for the majority of beneficial effects observed with MSCs in various disease models and early clinical trials through the action of secreted protein factors and microvesicles present in the conditioned medium [13,14,15,16,17]. Adipose-derived mesenchymal stromal cells (ASCs), and specifically the secretome products, have beneficial properties with improved metabolic stabilization and cell survival; reduced liver, kidney, lung, and muscle damage; and enhanced neuronal survival and protection against brain injury and inflammation [18,19,20,21,22]. These beneficial effects on multiple tissues and organs suggest that the MSC secretome could be a strategic point-of-injury regenerative therapeutic following TBI and hemorrhage.

Thera-101 (T-101) is a novel ASC secretome-based therapeutic developed by Theratome Bio, Inc. that is free of animal components and offers several potential competitive advantages over cellular therapy. The product is a liquid stored at −20 °C with a prototype lyophilized formulation in development, which would further ease administration in the pre-hospital environment. Additionally, T-101 contains a combination of freely soluble exosome-associated factors that have critical repair and regenerative therapeutic activity, including hepatocyte growth factor (HGF), vascular endothelial growth factor (VEGF), brain-derived neurotrophic factor (BDNF), nerve growth factor (NGF), and glial-derived neurotrophic factor (GDNF) [15,20,23,24]. Importantly, the administration of T-101 was effective in mitigating injury in a rat model of brain hypoxia-ischemia, leading to long-term improvements in motor and cognitive functions [24]. Thus, the beneficial properties of T-101 make it a promising candidate as a resuscitative fluid and multiplex repair therapeutic following TBI and hemorrhage.

In this study, T-101 was investigated in a poly-trauma rodent model of TBI and 35% volume-controlled hemorrhage to determine its efficacy in preventing and/or mitigating end organ damage, inflammation, and brain injury. Specifically, the effects of T-101 on systemic inflammation and liver, kidney, muscle, lung, and brain damage were evaluated as a therapeutic intervention to treat potentially survivable poly-trauma and reduce organ damage.

## 2. Results

### 2.1. Survival and Vitals

**Figure 1:**
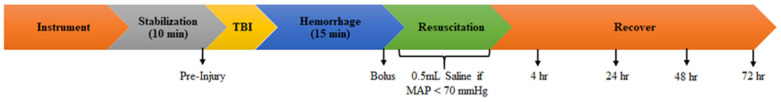
Injury timeline. Instrumentation was followed by 10 min of stabilization, at the end of which pre-injury vitals were recorded. TBI was completed, followed by the 35% volume-controlled hemorrhage. Resuscitation began with 1 mL of T-101 or the control (saline) resuscitative fluid group. If MAP remained below 70 mmHg for 5 min after 1 mL of resuscitative saline or T-101 fluid, then 0.5 mL of saline was infused at 0.1 mL/min. Then, cannulated vessels were ligated, catheters were removed, and the rat was recovered until endpoints at 4, 24, 48, or 72 h after the start of hemorrhage.

The overall study design layout is provided in Figure 1. Eight animals expired during the recovery period (starting after resuscitation) and before the endpoints. Of the eight animals that expired, three expired in the control (saline) resuscitative fluid group (one at 1 h, one at 1.3 h, and one at 2.5 h after resuscitation). Five animals expired in the T-101 resuscitative fluid group (one at 1.7 h, one at 2 h, one at 2.2 h, one at 3.6 h, and one at 7.2 h after resuscitation). There was no significant difference in survival between the control resuscitative fluid and T-101 resuscitative fluid groups (Figure S1). No incidence of skull fracture was observed. The sham group had zero mortality during the study.

Vitals, including heart rate, mean arterial pressure (MAP), saturation of peripheral oxygen (SpO_2_), and respiration rate, were monitored throughout the procedure and are listed in Table S1. As expected, MAP dropped following hemorrhage by 30–35 mmHg from pre-injury. Control resuscitative fluid and T-101 fluids returned MAP to pre-injury levels following the resuscitative bolus. The respiration rate increased after TBI and returned to pre-injury levels by the time the hemorrhage was complete. Changes in heart rate and SpO_2_ occurred throughout the procedure but did not reach significance. The animal was recovered and free to move about the cage following resuscitation, so vitals were not monitored throughout the recovery period. With regards to animals that required the additional 0.5 mL bolus of saline, a total of 6 out of the 23 animals in the control resuscitative fluid group, and 6 out of the 25 animals in the T-101 resuscitative fluid group required the additional 0.5 mL bolus of saline. Therefore, there were no significant differences in total resuscitative fluids required between the two groups.

### 2.2. Plasma Markers of Organ Damage and Inflammation

**Figure 2:**
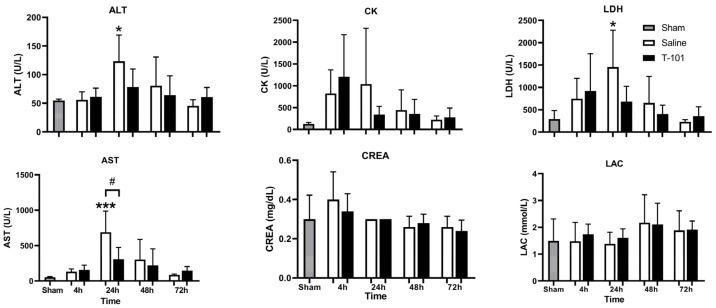
Blood chemistry plasma markers: alanine aminotransferase (ALT), aspartate aminotransferase (AST), creatine kinase (CK), creatinine (CREA), lactic acid dehydrogenase (LDH), and lactate (LAC). Data are plotted as the mean ± standard deviation. Significant differences for the control resuscitative fluid and T-101 groups when compared to the sham group are noted by an asterisk (* *p* < 0.05, *** *p* < 0.0005). Significant difference between the control resuscitative fluid and T-101 groups is noted by # (*p* < 0.05). Data compared by one-way ANOVA and the Bonferroni multiple comparison test. The analyzed groups included five animals (n = 5) per timepoint for the control and T-101 resuscitative fluid groups; five animals were analyzed in the sham group.

Blood chemistry was measured from plasma for the sham group and injury groups at 4, 24, 48, and 72 h after the start of hemorrhage (Figure 2). Aspartate aminotransferase (AST) and alanine aminotransferase (ALT) are enzyme markers of liver function. Elevated levels serve as liver damage indicators. The levels of ALT significantly (* *p* < 0.05) increased compared to the shams (54.8 ± 2.59 U/L) by 2.3-fold at 24 h in the control resuscitative fluid group (123.4 ± 45.73 U/L) while the T-101 group maintained ALT (4 h: 61.0 ± 15.44 U/L; 24 h: 78.2 ± 31.80 U/L; 48 h: 64.2 ± 33.80 U/L; 72 h: 60.8 ± 16.74 U/L) near sham levels at each timepoint. AST levels increased significantly (*p* < 0.05; 689.4 ± 299.29 U/L) at 24 h in the control resuscitative fluid group compared to the T-101 group (306.4 ± 169.61 U/L) and sham group (54 ± 7.48 U/L).

Creatine kinase (CK) is an enzyme that helps power muscle contraction. An increase in plasma levels is indicative of muscle damage. CK increased at 4 h for both injury groups and declined through to 72 h. The values were not significant due to the high variation between animals.

Lactic acid dehydrogenase (LDH) is a cellular enzyme that is rapidly released following plasma membrane damage, such as during necrosis or apoptosis. Elevated LDH is indicative of cell death. LDH initially peaked at 4 h in the T-101 group (not significant), and declined through to 72 h. The control resuscitative fluid group demonstrated a significant increase in LDH levels at 24 h (*p* < 0.05; 1455.8 ± 822.06 U/L) compared to the shams (293.2 ± 189.09 U/L). Both groups returned to near the sham levels by 72 h.

Creatinine (CREA) is a nitrogenous waste product and is used for kidney function evaluation. Elevated levels are indicative of kidney damage. CREA was increased at 4 h for both the control resuscitative fluid group and T-101, although this increase was not significant. CREA declined through to 72 h.

Elevated lactate (LAC) is indicative of activation of anaerobic metabolism and insufficient oxygen to tissues and organs. LAC levels were not significantly altered between groups.

**Figure 3:**
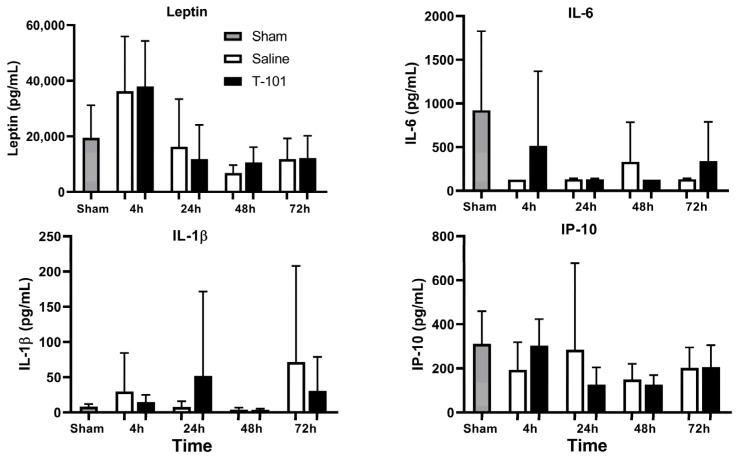
Systemic inflammation. Levels of leptin, interleukin-6 (IL-6), interleukin-1 beta (IL-1β), and inflammatory protein-10 (IP-10) were quantified from blood plasma collected for the sham group and injury groups at 4, 24, 48, and 72 h. Data are plotted as the mean ± standard deviation. There were no significant differences. Data compared by one-way ANOVA and the Bonferroni multiple comparison test. The analyzed groups included five animals (n = 5) per timepoint for the control and T-101 resuscitative fluid groups; five animals were analyzed in the sham group.

Plasma was also sampled to analyze systemic inflammation using the Luminex multiplex protein technology at 4, 24, 48, and 72 h after the start of hemorrhage (Figure 3). No significant differences between groups were noted for leptin, interleukin-6 (IL-6), interleukin-1 beta (IL-1β), or inflammatory protein-10 (IP-10) at the timepoints evaluated.

### 2.3. Histopathological Analysis of Organ Injury

**Figure 4:**
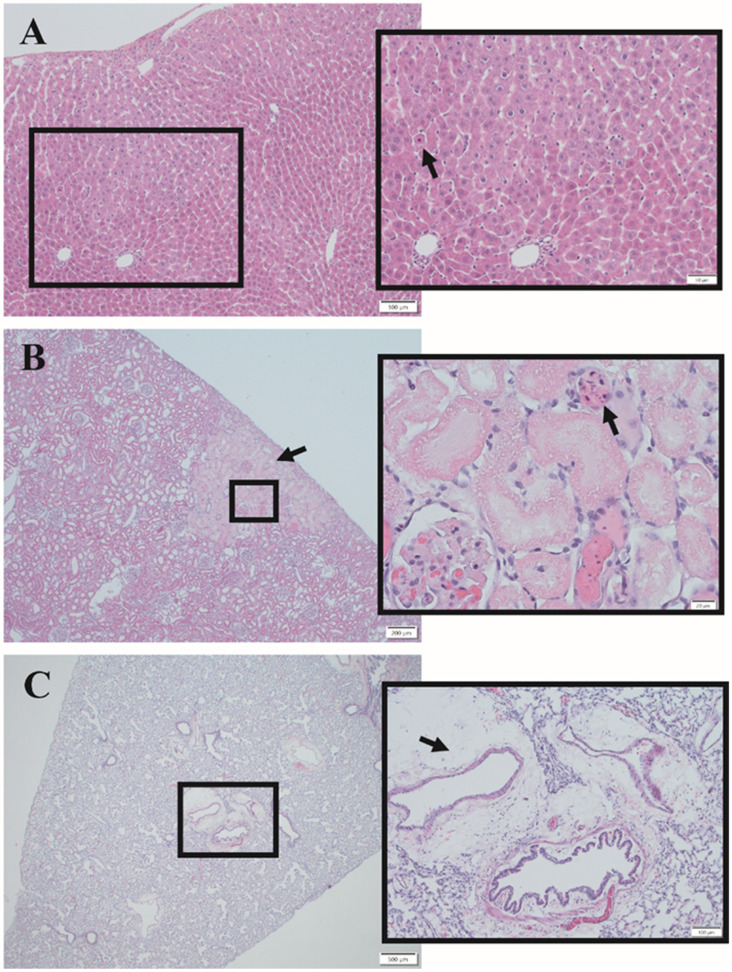
Hematoxylin and eosin histology Images. Representative images show histological findings in the (**A**) liver, (**B**) kidney, and (**C**) lung. Hepatocellular degeneration was observed in the liver with an arrow pointing to a necrotic hepatocyte in the control resuscitative fluid group (**A**). A focally extensive area of necrosis is indicated by the arrow. Renal tubular coagulative necrosis and rare lytic necrosis were observed as shown in the inset from the control resuscitative fluid group (**B**). Additional observations included intratubular casts of mucoprotein and glomerulus with marked mesangial and endocapillary fibrin, edema, and congestions admixed with increased hypercellularity, which is indicative of glomerulonephritis. Perivascular and vascular changes were observed in the lung for both groups; the image is from the T-101 group (**C**). The arrow points to mild to moderate multifocal perivascular edema. The inset shows one bronchiole and several large arterioles with moderate perivascular edema. Scale bars = (**A**) 100 µm, 50 µm; (**B**) 200 µm, 20 µm; (**C**) 500 µm, 100 µm.

The liver, kidney, and lung were evaluated by hematoxylin and eosin (H&E) staining to determine whether T-101 mitigated organ damage compared to the control resuscitative fluid. Parameters with significant differences from shams are identified with bold text in Table S2. Overall, the results indicated that T-101 imparted noted kidney protection (Figure 4). Specifically, renal necrosis was greater in the control resuscitative fluid group at 24 h (1.0 ± 0.9) compared to subjects that received T-101 (0.1 ± 0.3).

Additionally, the control resuscitative fluid group exhibited tubular degeneration that was significantly higher (*p* < 0.0005; 2.7 ± 0.5) than the sham group (0.00 ± 0.0) at 48 h while T-101 was minimally altered (0.09 ± 0.7). Effects on the liver were time dependent, with both groups exhibiting mild to moderate hepatic necrosis at four hours. Although the T-101 liver histopathology (dilation/fibrin and congestion) score showed a significant increase at 4 h (## *p* < 0.005; 1.6 ± 0.5) compared to the control resuscitative fluid group, the T-101 histopathology score returned to sham levels (0.0 ± 0) by 48 h. Additionally, the liver necrosis histopathology scores were consistently lower than the control resuscitative fluid group at all timepoints. Examination of the lungs showed that the control resuscitative fluid group exhibited perivascular edema at significantly higher levels (*p* < 0.05; 1.8 ± 0.8) compared to the sham group (0.00 ± 0) at 24 h and continued to increase, with significance observed again at 72 h (*p* < 0.005; 2.0 ± 0.7), compared to shams. The T-101 group exhibited a significant increase (*p* < 0.005; 2.8 ± 0.1) in perivascular edema at 4 h compared to the shams, which decreased to non-significant levels from 24 to 72 h. No significant differences between the control resuscitative fluid and T-101 groups were noted at any timepoint.

### 2.4. Evaluating the Neuroprotective Effects of T-101

**Figure 5:**
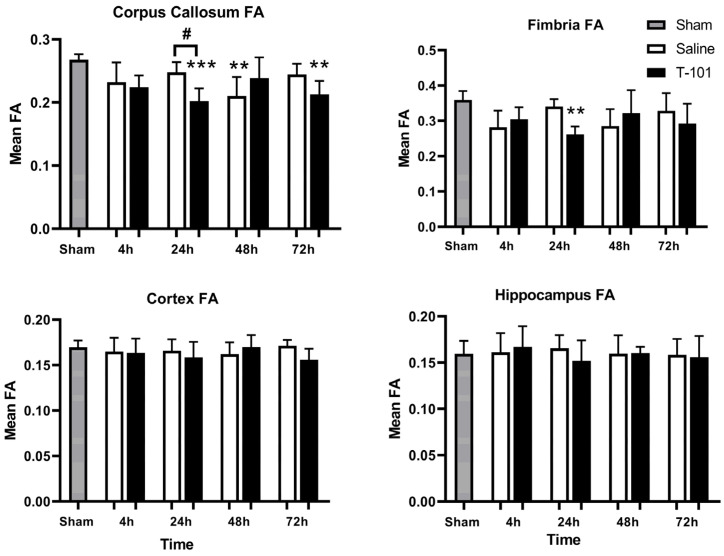
Diffusion tensor imaging (DTI) fractional anisotropy (FA) variation in brain regions. The greatest changes in FA were observed in the corpus callosum and the fimbria while there were no significant changes in the cortex or hippocampus. Data are plotted as the mean ± standard deviation, with significant differences from the sham for the control resuscitative fluid and T-101 groups noted by asterisks (** *p* < 0.005, *** *p* < 0.0005) and differences between injury groups for a given timepoint are noted by the pound sign (#, *p* < 0.05); Data compared by one-way ANOVA and the Bonferroni multiple comparison test. The analyzed groups included five animals (n = 5) per timepoint for the control and T-101 resuscitative fluid groups; five animals were analyzed in the sham group.

The neuroprotective effects of T-101 were evaluated by diffusion tensor imaging (DTI) and immunohistochemistry. First, the brain was scanned by magnetic resonance imaging (MRI) to obtain DTI maps. Fractional anisotropy (FA) was calculated from these DTI maps and lower values are indicative of injury with water diffusion in a direction other than along neuronal tracks. The greatest difference from the sham group was observed for FA in the corpus callosum followed by the fimbria (Figure 5). In the corpus callosum, FA for the control resuscitative fluid group was greater than T-101 at 24 and 72 h. This effect was reversed at 48 h, but there was also greater variability between subjects. The second most affected region of the brain was the fimbria. Similar to the corpus callosum, the greatest number of differences were noted in FA compared to the other DTI parameters, axial, radial, and mean diffusivity.

**Figure 6:**
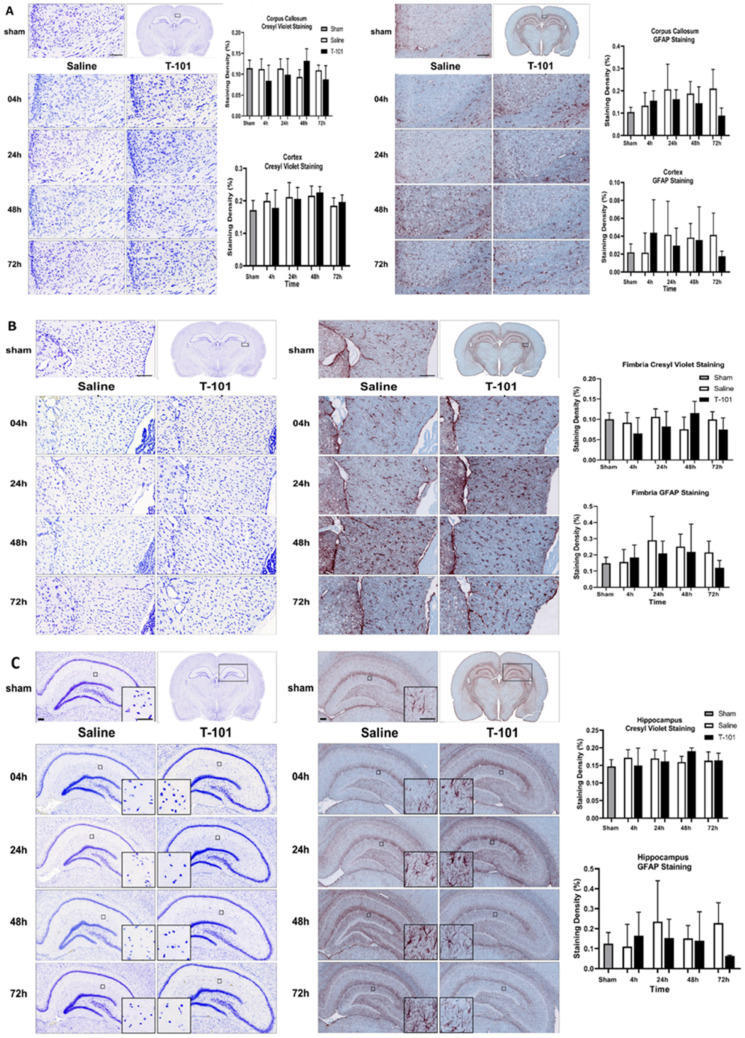
Brain immunohistochemistry. The (**A**) corpus callosum and cortex, (**B**) fimbria, and (**C**) hippocampus were stained with Cresyl Violet (left) for the Nissl substance in neurons and glial fibrillary acidic protein (GFAP) (right) for astrocytes. Data are plotted as the mean ± standard deviation; no significant differences between the SHAM and injury groups. Scale bar equals 100 µm. the analyzed groups included three animals per timepoint for the control and T-101 resuscitative fluid groups, and three animals were analyzed in the sham group.

Brain immunohistochemistry showed similar results between the sham and injury groups in the corpus callosum and overlying cortex, and the fimbria (Figure 6). Glial fibrillary acidic protein (GFAP) staining in the corpus callosum and fimbria of the T-101 group was generally lower than the control resuscitative fluid group from 24 to 72 h, with about a 50% reduction from the T-101 group mean compared to the control resuscitative fluid group mean, although these differences were not significant. In the hippocampus, there was increased Nissl substance for the T-101 group compared to the sham group and the control resuscitative fluid group at 48 h.

## 3. Discussion

As prolonged field care is anticipated in future conflicts, effective low-volume resuscitative fluids are critical to support our warfighters in future, resource-constrained environments. This report evaluated T-101, a therapeutic derived from the ASC secretome, to address this current gap. One goal of this study was to evaluate the ability of T-101 to reduce or mitigate organ damage. Blood chemistry results demonstrated that the model induced damage to the liver and muscle tissue and increased cell death at 4 and 24 h. Of these, T-101 had potential liver protection effects based on the blood chemistry results. Comparable trends in AST and ALT were observed in a similar injury model indicating that liver damage is common in poly-trauma with TBI and hemorrhage and therefore a focus for evaluating the efficacy of therapeutics [25]. Banas and colleagues reported that ASC whole-cell grafts improved liver function following injury and these benefits were attributed to secreted NGF, HGF, and VEGF [20]. It follows that T-101, abundant in these factors, would serve as a promising alternative while reducing the burdens of direct cellular administration. The most significant finding of the H&E staining results is the indication that T-101 may provide kidney protection, especially after 48 h. Histology results were similar to Ronn and colleagues, who reported pathological changes in the kidney, liver, and small intestine, but the lungs remained unaffected [26]. Chen et al. also reported that ASCs protected kidneys in an ischemia-reperfusion model by lowering oxidative stress and the inflammatory reaction [21]. Overall, the data suggests the potential robustness of secretome-based therapeutics to target multiple organs in a single drug.

The brain was evaluated by DTI and immunohistochemistry to evaluate injury severity and the effects of T-101. First, DTI was utilized as a tool to quantify brain injury, which was specifically attributed to diffuse axonal injury as is expected in this model. The degree of anisotropy in water diffusion along neuronal tracts in the brain revealed that the corpus callosum and fimbria were the most affected regions of the brain. Decreasing FA values indicate an injury, either due to dying neurons, damaged axons, or the surrounding myelin because the water diffusion has shifted to a radial plane [27,28]. In our study, we found no positive effects of T-101 on the mitigation of brain injury from TBI. Others have demonstrated the strong potential for a secretome-based therapy to mitigate brain damage as both the conditioned medium from ASCs and cell-based therapy improved motor and cognitive function in 6-month-old rats [22]. It is possible a larger dose of T-101 may be needed to mitigate brain injury from TBI.

Second, immunohistochemistry for each of the brain regions analyzed in this study showed changes after injury. Increased GFAP staining in the hippocampus and the bushy morphology and dense cell body suggest increased activation. While GFAP staining with T-101 treatment was generally less than the control resuscitative fluid group, it was not statistically significant. Similar trends were also observed in the corpus callosum and fimbria. T-101 could be reducing astrocyte activation in these regions, but due to the high variability, it was not statistically significant. Together, the imaging techniques used to evaluate the injury model and effects of T-101 showed trends toward lower astrocyte activation with T-101 treatment, but it is likely a higher dosage may be required for more clinically relevant differences. Outcomes were similar to other published reports on damage to the corpus callosum in TBI models [29]. The corpus callosum is the primary commissural region of the brain; the white matter tracts connecting the right and left hemispheres are responsible for transferring sensory, motor, and high-level cognitive signals [30]. Additionally, as the hippocampus is responsible for learning and memory, mitigating damage could have profound impacts on patient health [31]. Therefore, identifying a therapeutic that mitigates damage, particularly in this region of the brain, has the potential for significant clinical success.

We also assessed the potential impact of injury and T-101 on systemic inflammation over the course of 72 h. In our model, multiplexed analysis of inflammatory markers did not show a significant increase in leptin, IL-6, IL-1β, or IP-10 at any timepoint. These analytes were selected from a 27-plex inflammation panel as they resulted in detectable levels from initial pilot studies. In a similar injury model from a previous report, the treatment group also demonstrated similar IL-6 levels to shams four hours after injury, but levels were elevated in the control resuscitative fluid group [25]. In contrast, Xu et al. used a TBI rodent model followed by secretome dosing through day 7 that demonstrated reduced inflammation and apoptosis, with a noted change in microglia phenotypes from M1 cells (inflammatory) to M2 cells (anti-inflammatory). Inflammatory and anti-inflammatory mediators were evaluated through to day 14 post-TBI [32]. A separate study revealed that ASC-conditioned media enhanced protective neurovascular modulation in a mild traumatic brain injury model [33]. The consistency with sham levels in our model could be due to the timepoints when blood was collected or it could suggest that a more severe model might be necessary to observe this effect, which is often associated with poly-trauma. As the Sprague Dawley rats are an outbred strain, some variability is expected and could have attributed to the standard deviation with the groups.

In addition to the above-mentioned benefits of T-101, the ease of transitioning this therapeutic to the clinic is a significant advantage. T-101 is manufactured under good manufacturing practices (GMPs), which ensures stringent quality control measures are maintained for safe and consistent drug production. Additional benefits of secretome-based therapeutics include the multiplex repair factors encompassed in a single dose without the challenges of cellular delivery. However, few reports have quantified the levels of these factors so direct comparison between studies remains challenging. These factors have been defined and shown to be consistent across the T-101 lots during Theratome production. Since the therapeutic is derived from human cells, there is a lesser gap when transitioning to clinical studies, in contrast to animal-derived treatments. Therefore, risks associated with successful clinical transition are reduced.

## 4. Materials and Methods

### 4.1. Rat Poly-Trauma Model

This study protocol was approved by the Institutional Animal Care and Use Committee at the 711th Human Performance Wing, Joint Base San Antonio-Fort Sam Houston, in compliance with all applicable Federal regulations governing the protection of animals in research. This study is reported in accordance with the ARRIVE guidelines. The experiments reported herein were conducted in compliance with the Animal Welfare Act and per the principles set forth in the “Guide for Care and Use of Laboratory Animals”, Institute of Laboratory Animals Resources, National Research Council, National Academy Press, 2011.

Male, Sprague Dawley rats (290–325 g), acquired from Charles River Laboratories (Frederick, MD, USA), were acclimated to the facility for at least seven days. Subjects were pair-housed upon arrival and single-housed following the procedure on a 12-h light/dark cycle, with ad libitum access to food and water. Food and hydration support were placed on the floor of the cage following the procedure to ensure easy access during recovery. Each subject was induced with 3–3.5% isoflurane and administered a pre-operative dose of Buprenorphine HCl (0.1 mg/kg) (Reckitt Benckiser Healthcare, Slough, Berkshire, England). Anesthesia was maintained at 0.5–2% isoflurane and rats were placed on a physiological monitoring system (Harvard Apparatus, Holliston, MA, USA) that regulated body temperature and recorded heart rate (HR), peripheral oxygen saturation (SpO_2_), systolic and diastolic blood pressure, and respiration rate (RR).

The procedure timeline is depicted in Figure 1. First, vascular access was obtained by cannulating the femoral artery and vein with heparin-coated polyethylene (PE)-50 tubing (Instech Laboratories, Inc, Plymouth Meeting, PA, USA) and a rat femoral vein catheter (Instech Laboratories, Inc, Plymouth Meeting, PA, USA), respectively. Mean arterial pressure (MAP) was monitored with a pressure transducer (Micro-Med INC, Louisville, KY, USA) and Blood Pressure Analyzer (Micro-Med INC, Louisville, KY, USA) from the arterial line. The rat was moved to the prone position and a round metal disc (10 mm diameter by 3 mm thick) (online metals.com, Seattle, WA, USA) was centered and secured on the subject’s head using tissue glue. A 10 min stabilization period followed, then pre-injury vitals were recorded (Table S1). Next, the electrocardiogram leads and the SpO_2_ sensor were removed, the catheter lines were closed, and the subject was transferred to the TBI device consistent with the Marmarou weight drop model [34]. The device consisted of a platform with a 1.8-m-long clear plastic guide tube (22.23 mm outer diameter, 15.88 mm inner diameter) through which a 450 g brass weight fell freely. A foam bed (type E, Foam to Size, Ashland, VA, USA) was positioned inside a plexiglass container under the guide tube. TBI was induced by dropping the weight through the guide tube to allow contact with the metal disc on the rat’s head from a height of 1.25 m [34]. A velocity sensor (TDS 3054B, Tektronix, Beaverton, OR, USA) was used to ensure a consistent velocity of the weight near the bottom of the guide tube was achieved for each subject (5.0 ± 0.5 mm/s). Following TBI, the rodent was quickly transferred to the physiological monitoring system, the catheters were reconnected, and the ECG leads and SpO_2_ sensor were placed. Isoflurane was maintained at 1% for the duration of the procedure.

After obtaining a post-TBI MAP recording, the 35% volume-controlled hemorrhage was induced using a programmable syringe pump (Legato 110, KD Scientific, Holliston, MA, USA) at a withdrawal rate of 1 mL/min from the arterial catheter. The hemorrhage volume was calculated from the total estimated blood volume for rats at 64 mL/kg [35]. MAP was recorded immediately following the hemorrhage then monitored continuously throughout the remainder of the surgical procedure. Next, resuscitation was initiated 15 min after the start of hemorrhage [36]. Subjects were randomly assorted into groups to receive the control (saline) resuscitative fluid or the T-101 resuscitative fluid, with terminal end points at 4, 24, 48, or 72 h after the start of hemorrhage (n = 5/group at each timepoint required for analysis) according to the research design in Table S3. The number of animals used in this study was determined based on previous reports of brain and organ injury in rat models of TBI + hemorrhage [22,25]. The total number of rats used in this study, including the 8 rats that died before their designated timepoint, was 53 rats (5 sham rats, 23 injury +control resuscitative fluid rats (3 of which died early), and 25 injury + T-101 resuscitative fluid rats (5 of which died early)). A 1 mL bolus of control resuscitative fluid (saline) or 1 mL bolus of T-101 (1.7 mg/kg) resuscitative fluid was administered through the venous catheter using a T-101 dosage based on previous mouse stroke data [37]. An additional 0.5 mL of saline was infused at 0.1 ml/min if MAP did not reach 70 mmHg within 5 min of the bolus. Therefore, the maximum resuscitation volume was 1.5 mL per rat and resuscitation was completed within 10 min of the bolus. After resuscitation, the cannulated vessels were ligated, catheters and leads were removed, and a post-operative dose of Buprenorphine SR (1.0 mg/kg) (Zoopharm, Fort Collins, CO, USA) was administered. The animal was returned to a fresh cage for post-surgical monitoring until the endpoint was reached. Shams underwent all surgical preparation and were maintained under anesthesia for a period of time consistent with the control resuscitative fluid and T-101 groups, but they did not experience TBI or hemorrhage. The subjects were monitored for 72 h following the estimated time when hemorrhage would have been initiated, and then euthanized at the 72 h timepoint, with final blood collection and tissue harvesting at that 72 h timepoint.

At the assigned endpoint, all animals were placed under deep isoflurane anesthesia, given a dose of Buprenorphine HCl (0.1 mg/kg), and transcardial perfusion was performed as previously described [38]. A final blood draw was obtained immediately before the perfusion from the left ventricle and transferred to blood collection tubes with lithium heparin or ethylenediaminetetraacetic acid (EDTA) for blood chemistry or multiplexed analyte quantification, respectively. The perfusion was performed using the Leica Perfusion Two™ automated perfusion system (Leica Biosystems, Buffalo Grove, IL, USA) according to the manufacturer’s instructions by first infusing 10% sucrose to clear the blood, followed by 10% formalin for fixation.

### 4.2. Histopathology of Lung, Kidney, and Liver

The lungs, kidneys, and liver were transferred to 10% buffered formalin for at least 72 h and then embedded in paraffin (Fisherbrand^TM^ Histoplast PE, Pittsburg, PA, USA). They were then sectioned to a 5–6 µm thickness and stained with hematoxylin and eosin (H&E) stain (Hematoxylin+, Thermo Fisher Scientific, Pittsburg, PA, USA; Eosin-Y Alcoholic 0.25%, Stat Lab Medical Products, McKinney, TX, USA). One section per block was imaged and scored by a board-certified veterinary pathologist. Scores were assigned on a scale of 0–5 to indicate the severity or degree of histologic findings present in the examined tissue: 0 (not present), 1 (minimal), 2 (mild), 3 (moderate), 4 (marked), or 5 (severe). Cells were scored by the following scale: 0: negative; 1: <10% of cells in section are affected (minimal); 2: 11–25% of cells in section are affected (mild); 3: 26–50% of cells in section are affected (moderate); 4: 50%–75% of cells in section are affected (marked); and 5: >75% of cells in section are affected (severe).

### 4.3. Immunohistochemistry and Diffusion Tensor Imaging

For brain analysis, the subject’s head was fixed in 10% buffered formalin for 72 h then transferred to phosphate-buffered saline (PBS) containing 0.01% sodium azide for shipment to Biospective, Inc. (Montreal, QC, Canada) for ex vivo diffusion tensor imaging (DTI) and immunohistochemistry. Scientists at Biospective, Inc. remained blinded to the treatment group during data collection and analysis. Each brain specimen was evaluated by magnetic resonance imaging (MRI, 7T Bruker BioSpec 70/30 system, Bruker Biospin, Ettlingen, Germany). Samples were placed in the scanner and warmed to 44 ± 0.3 °C. Multi-dimensional, multi-shell images were obtained in 50 directions with 3 shells and 6 b0 images. The MRI images were processed with NIGHTWING^TM^ software (Biospective Inc, Montreal, QC, Canada). The lowest shell was removed from analysis due to ringing artifact. Each reconstructed image was corrected for non-uniformity with the N3 algorithm [39], brain masking, and linear spatial normalization with a 12-parameter affine transformation to map individual images from a native coordinate space to a reference space. Next, an unbiased, symmetric, anatomical template was generated from the b0 images [40,41] and images were linearly and nonlinearly registered to this anatomical template [40,42,43]. Image segmentation was achieved by identifying neuroanatomical regions on the atlas. DTI parametric maps were generated with an automated method to compute the mean diffusivity (MD), axial diffusivity (AD), radial diffusivity (RD), and fractional anisotropy (FA). The parametric maps were then mapped into the template space.

Following MRI, the brain tissue was prepared for immunohistochemistry. The brain specimens were extracted from the skull and processed for paraffin embedding. Blocks were sectioned in the coronal orientation at a thickness of 5 µm to target the anterior, middle, and posterior segments of the corpus callosum, overlying cortex, hippocampus, and fimbria. All sections were stained with Cresyl Violet to label the Nissl substance in neurons and immunostained for glial fibrillary acidic protein (GFAP) to label astrocytes. First, sections were de-paraffinized and rehydrated. The Cresyl Violet stain was prepared as a 0.1% stock of Cresyl Violet acetate (Sigma-Aldrich, St. Louis, MO, USA C5042) in deionized water. Ten drops of 10% acetic acid were added to a 30 mL stock solution for each round of staining. Slides were stained for three minutes, then dehydrated and mounted with Permount^TM^ mounting medium. GFAP staining was performed on a Lab Vision 360 Autostainer (Fisher Scientific, Toronto, ON, Canada) with detergent reagents from Abcam. The slides underwent epitope retrieval by incubating them in citrate buffer (pH 6.0) and heating to 120 °C under high pressure for 10 min. Endogenous peroxidase was quenched by sequential incubations in hydrogen peroxide for five minutes. Next, the slides were incubated in Protein Block (Abcam ab156024) for 5 min followed by 60 min with the primary antibody (rabbit Ab anti-GFAP, Thermo Fisher RB-087-A; 1:100). The secondary antibody (donkey anti-rabbit IgG (Jackson ImmunoResearch, West Grove, PA, USA) and Streptavidin-HRP (Abcam, Waltham MA, USA ab64269)) conjugate was added for tissue visualization using AEC Single Solution (Abcam, Waltham MA, USA ab64252) for 20 min. The sections were counterstained with Acid Blue 129 (Sigma-Aldrich, St. Louis, MO, USA) and mounted with aqueous mounting medium [44]. The stained sections were digitalized on an AxioScan.Z1 digital whole slide scanner (Carl Zeiss, Toronto, ON, Canada). Image processing involved manually delineating regions of interest and using the automated PERMITS^TM^ quantification process to determine the percent positive-stained area.

### 4.4. Laboratory Assays

Blood plasma collected in lithium heparin tubes was used for blood chemistries. The IDEXX Catalyst One Analyzer (IDEXX, Westbrook, Maine, USA) clips included creatinine (CREA), creatine kinase (CK), alanine aminotransferase (ALT), aspartate aminotransferase (AST), lactate (LAC), and lactate dehydrogenase (LDH). Blood plasma specimens collected in EDTA tubes were evaluated for inflammatory markers. Initially, a 27-plex assay was evaluated to identify detectable analytes to down-select. Next, a customized rat four-plex cytokine/chemokine kit (Millipore, Burlington, MA, USA) on a Bio-plex^®^ 200 Luminex system (Bio-Rad, Hercules, CA, USA) was used for analyses according to the manufacturer’s instructions based on the analytes that had detectable levels from the 27-plex assay. The custom kit included interleukin (IL)-1β, IL-6, inflammatory protein-10 (IP-10), and leptin.

### 4.5. Statistical Analysis

Data are plotted as the mean ± standard deviation and statistics were run on GraphPad Prism 8.3 (GraphPad Software, San Diego, CA, USA), with *p* < 0.05 considered to be statistically different. Vitals data were analyzed with repeated-measures ANOVA. The analysis for blood chemistry, Luminex, histology, and brain assessment were performed on n = 5 rats/group that survived to their endpoint. Log-transformed blood chemistry and inflammation data were analyzed with a one-way ANOVA comparing injury groups and the sham group, with the Bonferroni multiple comparison test. Histological assessment scores were analyzed for each group compared to the Sham group via the one-way ANOVA Kruskal–Wallis test with Dunnett’s multiple comparisons post hoc test. All brain analysis data were analyzed using a one-way ANOVA and the Bonferroni multiple comparison test. Voxel-wise statistical analyses of the DTI maps were completed using the SurfStat toolbox. The *p*-values without correction for multiple comparisons were reported and *p* < 0.05 were considered significant.

## 5. Conclusions

Overall, results indicate T-101 may have the potential to serve as a protective or reparative resuscitative fluid to reduce liver and kidney organ damage based on reduced AST and ALT levels and improved viability of renal cells compared to the control resuscitative fluid group, as shown by pathology analyses. Further investigations and formulation refinement are required to probe the systemic response and establish the optimal dosing and formulation regimen in a model of increased severity. Future studies will also focus on assessing the efficacy of T-101 on TBI in not only the sub-acute stage, which this study focused on, but beyond 72 h post-injury. Additional therapeutic delivery regimens and behavioral evaluation to further assess regenerative aspects will promote improved quality of life following poly-trauma. A lyophilized formulation of T-101 is in development, which allows for a field-deployable therapeutic without <0 °C storage conditions. Further refinement and formulation optimization of this therapeutic could provide military and civilian first responders with an intervention to treat poly-trauma injuries and improve survivable injury outcomes.

## Data Availability

The datasets generated and/or analyzed during the current study are available from the corresponding authors upon request.

## Supplementary Materials

The following supporting information can be downloaded at: https://www.mdpi.com/article/10.3390/ijms232012664/s1.
