# Supplementary material for: Evaluating Thera-101 as a Low-Volume Resuscitation Fluid in a Model of Polytrauma
Source: Int J Mol Sci. 2022 Oct 21;23(20):12664. doi: 10.3390/ijms232012664 (PMC9604349; doi:10.3390/ijms232012664)
Supplement: Supplementary file 1 [file ijms-23-12664-s001.zip › ijms-1929861-supplementary.pdf]

Supplementary Material

Figure S1: Survival; Table S1: Vitals; Table S2: Pathology; Table S3: Research Design.

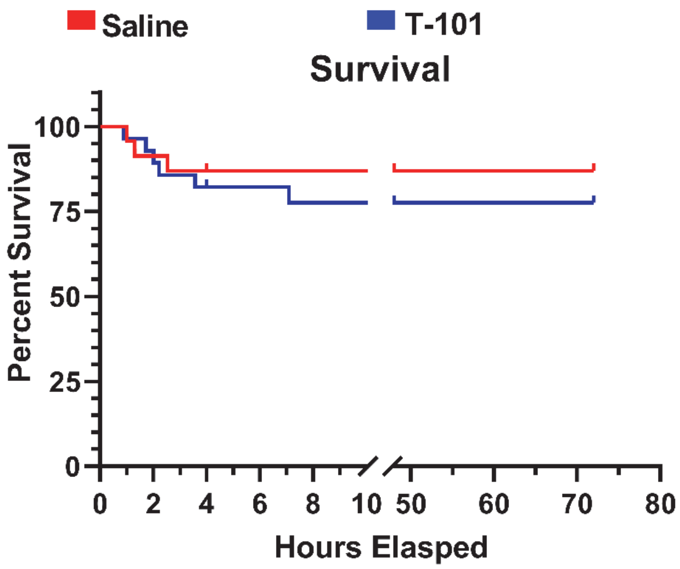

Figure S1. Kaplan-Meier Survival Curve. No significant differences were observed between the control resuscitative fluid (Saline) and T-101 groups. Eight animals expired during the recovery period and before the endpoints, three animals in the control resuscitative fluid group and five animals in the T-101 resuscitative fluid group. The Sham group had zero mortality during the study.

Table S1. Vitals. Vitals are reported as mean ± standard deviation for heart rate (HR), Mean arterial pressure (MAP), saturation of peripheral oxygen (SpO_2), and respiration rate (RR) at key points throughout the protocol in the control and T-101 resuscitative fluid groups are shown. Significant differences from Pre-Injury are noted in bold text.

Vital	Group	Pre-Injury	After TBI	After Hemorrhage	After Bolus	End of Resuscitation
HR	Vehicle	316.2±34.2	307.4±48.2	329.1±46.3	326.0±36.4	339.4 ±39.7
	T-101	311.7±72.0	294.3±89.3	321.3±81.8	325.5±40.7	345.8±85.6
MAP	Vehicle	86.9±6.1	**70.9±14.3**	**56.5±15.6**	80.6±10.0	92.5±11.7
	T-101	89.2±9.6	73.2±17.3	**52.2±19.3**	85.9±20.0	89.1±17.1
SpO_2	Vehicle	97.3±3.0	95.9±3.8	93.9±4.4	96.3±2.7	95.4±7.7
	T-101	96.8±20.6	96.9±20.5	94.1±20.5	96.3±3.8	96.2±20.4
RR	Vehicle	68.8±22.7	75.6±17.7	64.3±22.7	61.0±15.6	73.2±24.3
	T-101	60.9±17.6	**81.7±22.2**	60.8±24.0	64.6±16.1	76.2±26.8

Table S2. Histological analysis of the kidney, liver, and lung. Data are reported as mean ± standard deviation at each time-point for rats receiving the vehicle control or T-101. Significant differences from Shams are shown in bold text (**P*<0.05, ***P*<0.005, *** *P*<0.0005). Significant differences between the control and T-101 resuscitative fluid groups are shown with symbols (##: *P*<0.005).

	Group	4h	24h	48h	72h
**Liver**					
Dilation/ Fibrin & Congestion with reactive endothelium portal and centrilobular veins only	**Vehicle**	0.0+/-0.0	0.2+/-0.4	0.0+/-0.0	0.0+/-0.0
	**T-101**	**1.6+/0.5##/****	**1.2+/-0.4****	0.2+/-0.4	0.0+/-0.0
	**Shams**	0.0+/-0.0			
Hepatic degeneration	**Vehicle**	1.4+/-0.5	2.0+/-0.7	1.6+/-1.3	0.4+/-0.5
	**T-101**	1.4+/-0.5	0.5+/-0.5	1.6+/-0.9	1.2+/-2.1
	**Shams**	0.6+/-1.3			
Hepatic necrosis	**Vehicle**	0.4+/-0.5	0.8+/-0.4	1.0+/-1.2	0.4+/-0.5
	**T-101**	0.2+/-0.4	0.0+/-0.0	0.2+/-0.4	0.0+/-0.0
	**Shams**	0.0+/-0.0			
**Kidney**					
Fibrin/Edema, Glomerular Tufts	**Vehicle**	0.4+/-0.5	**3.3+/-0.5*****	**3.1+/-0.7****	1.2+/-0.9
	**T-101**	1.1+/-0.7	**2.9+/-0.5****	1.4+/-0.7	1.6+/-0.7
	**Shams**	0.0+/-0.0			
Glomerularsclerosis/ Glomerulonephritis	**Vehicle**	1.3+/-1.16	**3.2+/-0.4****	**3.0+/-0.7***	1.0+/-0.9
	**T-101**	**2.5+/-1.1***	**2.9+/-0.5****	0.7+/-0.7	1.0+/-0.8
	**Shams**	0.0+/-0.0			
Tubular Necrosis (Coagulative, Single cell, etc.)	**Vehicle**	0.0+/-0.0	1.0+/-0.9	0.1+/-0.3	0.0+/-0.0
	**T-101**	0.0+/-0.0	0.1+/-0.3	0.0+/-0.0	0.0+/-0.0
	**Shams**	0.0+/-0.0			
Tubular Degeneration	**Vehicle**	2.0+/-1.1	**3.3+/-0.5*****	**2.7+/-0.5***	0.3+/-0.5
	**T-101**	**2.3+/-0.5***	**3.1+/-0.5*****	0.9+/-0.7	0.3+/-0.5
	**Shams**	0.0+/-0.0			
**Lung**					
Edema/fibrin/+/-hemorrhage, Subpleural	**Vehicle**	0.2+/-0.4	**0**.4+/-0.5	0.6+/-0.5	0.6+/-0.5
	**T-101**	1.0+/-0.7	0.7+/-0.8	0.8+/-0.4	1.2+/-0.4
	**Shams**	0.2+/-0.4			
Edema/fibrin, Perivascular	**Vehicle**	1.6+/-0.5	**1.8+/-0.8***	1.8+/-1.3	**2.0+/-0.7****
	**T-101**	**2.8+/-1.1*****	1.7+/-0.8	1.4+/-0.5	1.2+/-0.4
	**Shams**	0.0+/-0.0			
Hemorrhage, Perivascular	**Vehicle**	1.2+/-1.3	0.6+/-0.5	0.2+/-0.4	0.0+/-0.0
	**T-101**	1.2+/-1.3	0.0+/-0.0	0.4+/-0.5	0.4+/-0.5
	**Shams**	0.0+/-0.0			
Edema/fibrin/+/-hemorrhage, Alveolar	**Vehicle**	1.4+/-1.1	1.2+/-0.4	1.4+/-0.5	0.6+/-0.9
	**T-101**	1.6+/-1.5	1.2+/-1.0	1.0+/-0.0	1.2+/-0.4
	**Shams**	0.2+/-0.4			
Congestion, Alveolar Septa (with fibrin/edema)	**Vehicle**	0.8+/-0.8	1.4+/-1.1	1.2+/-1.1	0.6+/-0.9
	**T-101**	1.8+/-1.3	2.7+/-0.8	1.2+/-0.8	2.0+/-0.7
	**Shams**	0.8+/-0.8			

Table S3. Research Design. The table below shows the grouping of the animals for the research design. All injury rats analyzed (n=40) were subjected to a polytrauma which consisted of a brain injury induced by weight drop, followed by a 35% volume-controlled hemorrhage. Sham rats (n=5) were cannulated and anesthetized, but did not receive any injury. They were survived out to 72hr before euthanasia and analysis. The injury rats were divided into two main groups of vehicle control resuscitative fluid, or T-101 resuscitative fluid. Those two treatment groups were further subdivided into time-point subgroups of 4hr, 24hr, 48hr or 72hr, which is the time-point that the injury rats were euthanized at, after injury and analyzed for all readouts.

Group	4hr	24hr	48hr	72hr
Sham	---	---	---	N=5
Vehicle	N=5	N=5	N=5	N=5
T101	N=5	N=5	N=5	N=5
